# EEG Features of Evoked Tactile Sensation: Two Cases Study

**DOI:** 10.3389/fnhum.2022.904216

**Published:** 2022-06-09

**Authors:** Changyu Qin, Wenyuan Liang, Dian Xie, Sheng Bi, Chih-Hong Chou

**Affiliations:** ^1^National Research Center for Rehabilitation Technical Aids, Beijing, China; ^2^Beijing Key Laboratory of Rehabilitation Technical Aids for Old-Age Disability, Beijing, China; ^3^Beijing Language and Culture University, Beijing, China; ^4^School of Biomedical Engineering, Shanghai Jiaotong University, Shanghai, China

**Keywords:** evoked tactile sensation, somatosensory evoked potential, prosthetic hands, projected finger map, EEG

## Abstract

**Purpose:** Sensory feedback for prosthetics is an important issue. The area of forearm stump skin that has evoked tactile sensation (ETS) of fingers is defined as the projected finger map (PFM), and the area close to the PFM region that does not have ETS is defined as the non-projected finger map (NPFM). Previous studies have confirmed that ETS can restore the tactile pathway of the lost finger, which was induced by stimulation of transcutaneous electrical nerve stimulation (TENS) on the end of stump skin. This study aims to reveal EEG features between the PFM and the NPFM regions of the stumps under the same TENS stimulation condition.

**Methods:** The PFM and NPFM regions of the two subjects were stimulated with the same intensity of TENS, respectively. TENS as target stimuli are modulated according to the Oddball paradigm to evoke the P300 components.

**Result:** The PFM regions of both subjects were able to elicit P300 components, while their NPFM regions were not able to elicit P300 components. However, this P300 appears early (249 ms for subject 1,230 ms for subject 2) and has continuous positive peaks (peak 1,139 ± 3 ms, peak 2,194 ± 0.5 ms) in front of it.

**Discussion:** N30 and P300 can prove that the two subjects with PFM can perceive and recognize ETS. The heteromorphisms of the P300 waveform may be related to the difficulty in subjects’ cognition of ETS or caused by the fusion of P150, P200, and P300.

## Introduction

Sensory feedback was considered to be the most lacking function of the upper prosthesis (Antfolk et al., 2010; D’Anna et al., 2017; Raspopovic et al., 2021). The reason for this phenomenon is that the current prosthesis lacks a suitable method to establish an effective and sensitive sensory feedback pathway for amputees. The tactile feedback pathway is a delicate and complex project that includes receptors, the nerve conduction pathway, and the central sensory nervous system. Normal people’s hands can feel touch primarily because the mechanoreceptors on the surface of the finger skin convert stimuli into electrical signals (Mountcastle, 2005), but amputees have lost the mechanoreceptors of their fingers due to surgery. Therefore, how to enable amputees to restore fine and anthropomorphic sensations is one of the important issues that need to be addressed in prosthetic sensory feedback technology.

Some scholars have proposed implanting invasive electrodes in the stump and stimulating the radial, ulnar, and median nerves to solve the sensory feedback problem (Raspopovic et al., 2014; Tan et al., 2014; Oddo et al., 2016), but this solution has the risk of infection that will not be accepted by most amputees. Other researchers offer a non-invasive sensory feedback solution, ETS, which was evoked by stimulation in the PFM region. The types of stimulation could be mechanical stimulation or TENS. Furthermore, studies have indicated that amputees can feel a more natural and richer tactile sensation under TENS than under mechanical stimulation (Mulvey et al., 2012; Zhang et al., 2022).

Research on ETS provides a new idea for sensory feedback-type prostheses. Although some scientists have successively studied and confirmed the richness and controllability of ETS (Chai et al., 2015; Li et al., 2018), there are not many studies on the characteristics of ETS in the EEG. EEG was one of the most convenient and reliable methods to study cognitive activity (Klimesch, 1999; Antonenko et al., 2010). Therefore, this article will use EEG to study the relevant characteristics of ETS.

## Materials and Methods

### Participants

This study was approved by the local ethics committee and all subjects signed a consent form. Two amputee participants were recruited from the National Research Center for Rehabilitation Technical Aids in Beijing, China. For recruiting principles, first, recruiting amputees should not have taken drugs in recent 3 months; second, the level of amputation is at the forearm level; third, amputees with PFM can evoke a tactile sensation of the fingers under mechanical pressure in the PFM area. Subject 1 (male, 28 years) was amputated in the middle and posterior of the right forearm 17 months ago in a burn accident. Subject 2 (male, 27 years) was amputated in the middle right forearm caused by a blast accident 15 years ago. Both subjects reported that their lost tactile sensation of the finger was evoked in the PFM area, where they were stimulated by mechanical stimulation and TENS. Both subjects have a clear evoked tactile sensation (ETS) of the middle finger in each PFM area. The NPFM region of subject 1 does not have specific feelings under any stimulation, while the NPFM region of subject 2 had the sense of touch on the skin surface. It needs to be stated that the sense of touch in subject 2’s NPFM region is different from the sense of touch of the evoked finger tactile sensation.

### Stimulation and EEG Acquisition Equipment

**Figure 1:**
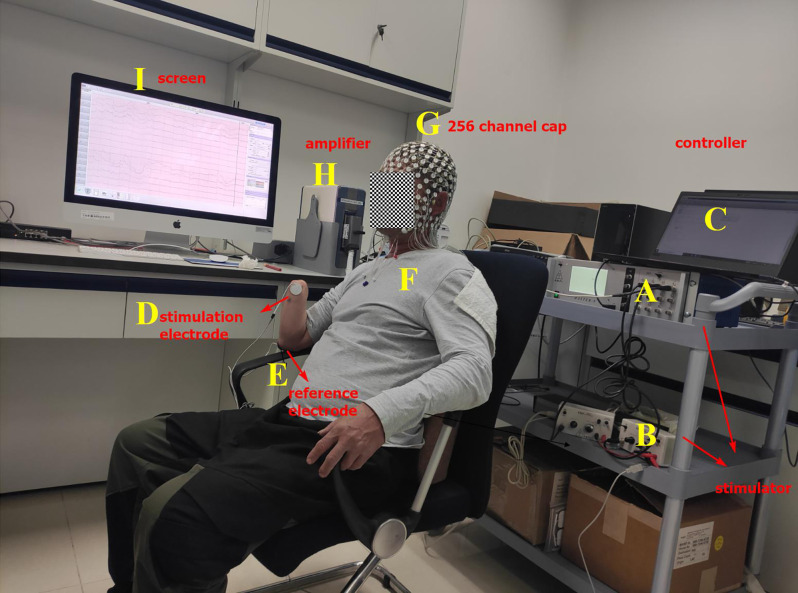
Experimental platform. **(A)** Master-9, programmable electrical stimulation device. **(B)** Two isolators. **(C)** Computer. **(D)** Stimulation electrode that is glued to the projected finger map (PFM) region. **(E)** Reference electrode that is glued to the olecranon. **(F)** Amputee. **(G)** EEG cap with 256 channels. **(H)** EEG signal amplifier. **(I)** Screen.

The electrical stimulation device, which is produced by AMPI Company, Israel, is composed of master-9 (Figure 1A) and two isolators (Figure 1B). The stimulation device is controlled by a computer (Figure 1C). Master-9 and two isolators can produce biphasic and charged-balanced current pulses. To find the infimum and supremum thresholds of finger ETS for each PFM, the current pulse amplitude was modulated at a pace of 1 mA ranging from 5 mA to 15 mA. The pulse-width was fixed to 200 μs.

**Figure 2:**
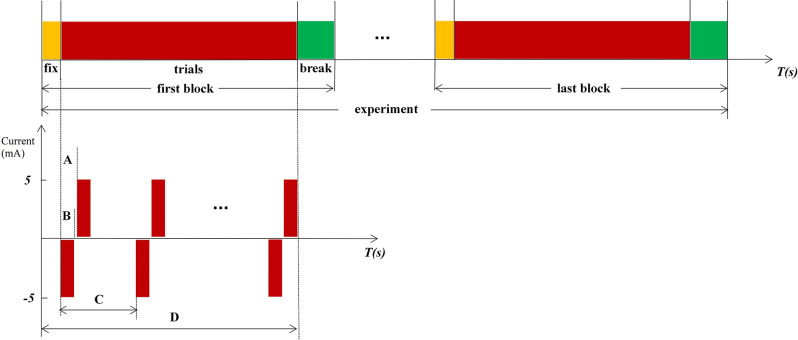
Experimental procedure and paradigm. **(A)** Internal pulse interval, 220 μs. **(B)** Pulse width, 200 μs. **(C)** The interval between each set of biphasic, a random value between 8 and12, and the unit is second. **(D)** Time of 1 fix and 15 stimulations.

When the ETS thresholds for each PFM are found, the intensity of the stimulation current is set to 1.5 times the infimum threshold, where the stimulation current is less than the supremum threshold. The stimulation frequency was randomly changed from 1/8 to 1/12 Hz. Therefore, the interval between each set of biphasic was 8–12 s (Figure 2C). Two isolators emit currents that stimulate the PFM region to evoke a tactile sensation of the finger using two electrodes (circle, 2 cm in diameter), where one electrode serves as the stimulation electrode (Figure 1D) and is glued to the PFM region, and the other as a reference electrode (Figure 1E) is placed on the olecranon. Subjects (Figure 1F) were asked to count (Desmedt and Tomberg, 1989) when they felt stimulated.

The EEG acquisition device (Figures 1G–I) is the EGI system (EGI, EGI Company, USA). In order to reduce the computation cost, 150 scalp channels that cover the whole scalp are extracted from 256 channels that cover the scalp, face, and neck for analysis. The original sampling rate is 1 kHz.

### Experimental Procedure and Paradigm

Stimulation is based on the Oddball paradigm, which requires subjects to click the mouse or count when lower probability stimuli < 20% appeared. In this experiment, the standard stimuli (frequent stimuli) are blank without any electrical stimulation, and the target stimuli (rare stimuli) are random electrical stimulations. The count was used as a response method in our experiment. Subjects were requested to participate in three experiments, stimulations in the PFM region, NPFM region, and the contralateral healthy middle finger, respectively. Both subjects reported the same type and intensity of middle finger sensation when electrical stimulation stimulated PFM regions and the contralateral healthy middle fingers. Due to the small sample size, we collected multiple sets of data for each sample to enrich the analysis data. Each experiment includes 150 trials, which were divided into 10 blocks. Subjects can fix before every block starts and have a break when the block ends (Figure 2). The subjects cannot predict the next stimulus because the stimulation interval is ranged from 8 to 12 s randomly. In Figure 2, the negative phase of each electrical stimulation is the effective component of stimuli, and the positive phase of each electrical stimulation is used to balance the electric charge of the skin surface.

### EEG Processing

The data were analyzed using Matlab (The Mathworks, USA). The ground electrode is the COM electrode. The reference electrodes (#94 and #190) are bilateral mastoid electrodes, corresponding to the TP9 and TP10 electrodes in the 10–20 system. The notch filter, with a lower edge of 49 Hz and a higher edge of 51 Hz, is adopted to reduce power frequency interference. Preprocessing includes baseline correction, bandpass filtering (1–30 Hz), epoch (-200–1,000 ms), and independent component analysis (ICA) which optimize artifact rejection. Draw butterfly figure to plot time-domain diagram of 150 channels and topography of peak (Figures 4A–D). Overlap the ERPs of each trial to plot the average ERP of Cz (Figures 4E,F).

**Figure 3:**
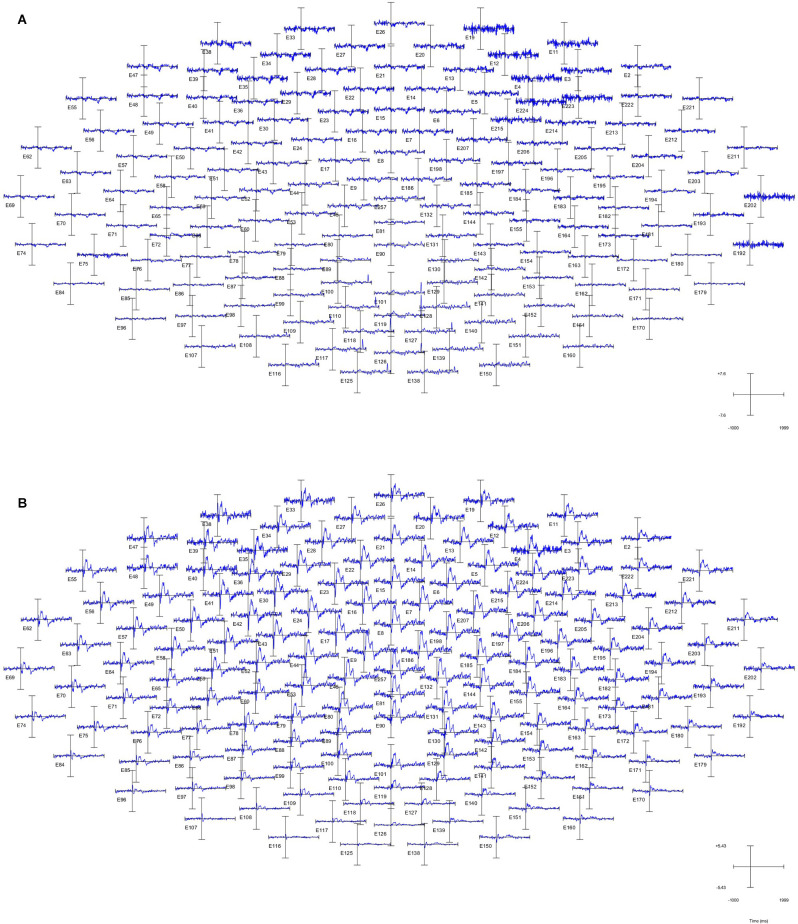
Time-domaindiagram of the whole brain of subject 1. **(A)** Non-projected finger map (NPFM) was stimulated. **(B)** PFM was stimulated.

**Figure 4:**
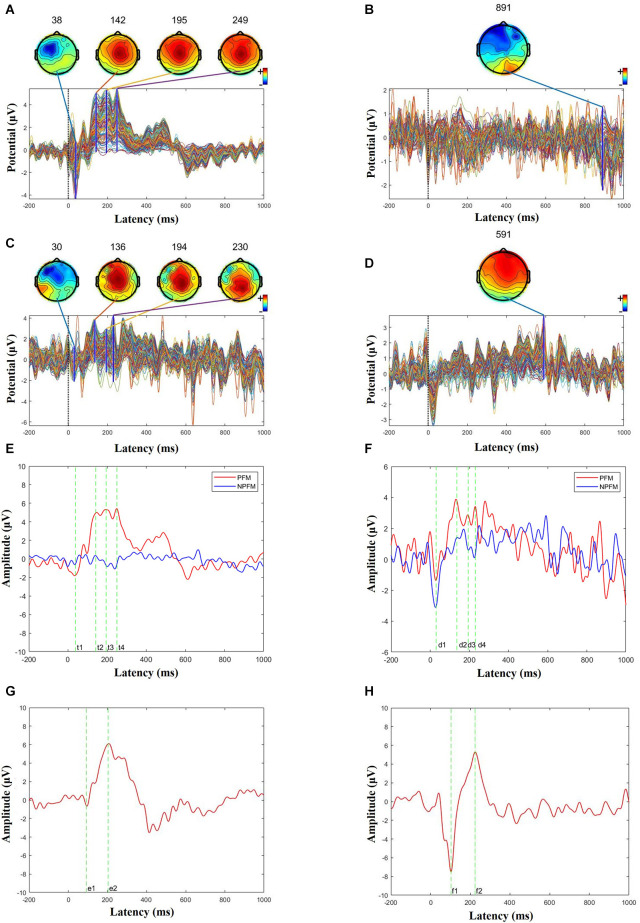
Butterfly and ERP maps. **(A)** PFM butterfly map of subject 1. **(B)** NPFM butterfly map of subject 1. **(C)** PFM butterfly map of subject 2. **(D)** NPFM butterfly map of subject 2. **(E)** Superimposed PFM and NPFMERP figure of subject 1 (*t*^1^ = 38 ms, *t*^2^ = 142 ms, *t*^3^ = 195 ms, *t*^4^ = 249 ms). **(F)** Superimposed PFM and NPFM ERP figure of subject 2 (*d*^1^ = 30 ms, *d*^2^ = 136 ms, *d*^3^ = 194 ms, *d*^4^ = 230 ms). **(G)** Superimposed healthy finger ERP figure of subject 1 (*e*^1^ = 93 ms, *e*^2^ = 204 ms). **(H)** Superimposed healthy finger ERP figure of subject 2 (*f*^1^ = 103 ms, *f*^2^ = 224 ms).

## Results

ERP (event-related potential) of the whole-brain can characterize the intensity of activity in every region of the brain. The high-density electrode system of the EGI has a higher spatial resolution than the conventional 10–20 system. As shown in Figure 3, the PFM region stimulated by TENS evoked obvious ERP waveforms (Figure 3B), while the NPFM region did not (Figure 3A). In Figure 3, pictures of **(A)** and **(B)** represent the average of the 150 trials. In addition, as the waveform shows, the intensity of the central region is greater than that of the surrounding area, and the signal in the occipital lobe is weakest.

Choose the Cz channel of the cap as the analysis channel, since Cz has the strongest SEP (somatosensory evoked potential) signal (Valeriani et al., 2000), and in this article, Cz also has the strongest potential (see Figure 3B). The largest superimposed peaks on the topographic maps are 249 ms in Figure 4A when the PFM of subject 1 is stimulated, 891 ms in Figure 4B when the NPFM of subject 1 is stimulated, 230 ms in Figure 4C when the PFM of subject 2 is stimulated, and 591 ms in Figure 4D when the NPFM of subject 2 is stimulated. Obviously, peaks of 891 ms in Figure 4B and 591 ms in Figure 4D are not be evoked by TENS. Furthermore, peaks of 38 ms, 142 ms, 195 ms in Figure 4A and peaks of 30 ms, 136 ms, and 194 ms in Figure 4C are selected to plot the butterfly map because their grand averaged values have greater amplitude in Figures 4E,F.

The PFM regions of the two subjects evoked the largest peaks at 249 ms (see Figure 4E) and 230 ms (see Figure 4F), respectively. As shown in Figures 4A–C, they shared similarities in intensity and topography with the highest amplitude in Cz and symmetric distribution. More details about P150 and P200 will be shown in the next section.

N30 evoked by TENS in both PFM showed the contralateral frontal distribution on the map, especially N30 was also induced in the NPFM of subject 2 (Figure 4F), which could be related to what he said about the sensation of the skin surface under TENS.

In contrast, the ERPs of the contralateral healthy middle fingers are shown in Figures 4G,H. For Subject 1, the ERP has a negative peak at 93 ms and a positive peak at 204 ms, respectively (see Figure 4G). For Subject 2, the ERP has a negative peak at 103 m and a positive peak at 224 ms, respectively (see Figure 4H). The positive peaks in the ERPs of the contralateral healthy middle fingers decline rapidly and do not generate several continuous peaks compared to the ERPs of the PFM regions.

## Discussion

This study mainly describes the characteristics of ETSSEP in the PFM area stimulated by TENS in two amputees. The evoked finger tactile sensation signal in PFM will be uploaded to the primary sensory area S1 of the brain through the spinothalamic tract. Therefore, N30 can actually reflect the integrity of the pathway. The character of the N30 topography map shows contralateral brain activation as the right stump is stimulated (see Figures 4A–C).

### Cognitive Components Induced by Stochastic Low-Frequency Stimuli

The stimulation frequency determines the evoked components of EEG. The higher the stimulation frequency, the more obvious the short-latency components. Therefore, a commonly used stimulus paradigm called steady-state somatosensory evoked potentials (SSEPs; Hebert et al., 2014; Overstreet et al., 2019) evoked by stable and high-frequency ( > 5 Hz) stimulation, is often used as a diagnostic method for neurological diseases due to rarely affected by cognition. There are currently two reports using SSEPs to study ETS. Wang et al. (2021) used EEG to analyze a subject with amputation and showed the EEG characteristics of different stimulation sites; Su et al. (2020) initially studied the amplitude of SSEPs as an evaluation indicator of feeling. Unlike previous studies, the Oddball paradigm used in this article is to investigate the main cognitive component P300 (Kanda et al., 1996). Therefore, this research lowers the stimulation frequency and randomizes the inter-stimulation interval. Fortunately, P300 of this experiment appeared at 249 ms and 230 ms. Furthermore, a flat high potential distribution in the 150 ms–400 ms interval, called “plateau”, differs from previous SEP research (Akyüz et al., 1995; Artoni et al., 2020).

### Short- and Long-Latency Component Analysis

In this experiment, the negative wave peak components are evoked at 38 ms and 30 ms when stimulating the PFM areas of two subjects, respectively, which can be named N30 according to their latency. Some SEP experiments of median nerve stimulated by current also produced N30 components (Kaňovský et al., 2003) which were generated in the motor cortex (Waberski et al., 1999; Balzamo et al., 2004), and the Parkinson’s disease (PD) population had lower N30 amplitudes than normal individuals (Pierantozzi et al., 2000). Obviously, the N30 representing sensorimotor integration (Waberski et al., 1999; Pierantozzi et al., 2000; Balzamo et al., 2004) is not the same as the N30 in this experiment.

N30 in this article is somewhat similar to the N20 (Hlushchuk and Hari, 2006) of the SEP evoked by electrical stimulation of the median nerve because both topographic maps feature activation of the stimulated contralateral brain area, which represents signal transmission to the area S1. N20 is the earliest cortical processing in the primary somatosensory cortex (Passmore et al., 2014). The reason for the delay of N20 latency may be related to stimulation conditions. The latency of N20 is related to the arm length of the subjects (Desmedt and Tomberg, 1989), so it cannot be ruled out that the difference between TENS stimulation and median nerve stimulation causes the delay.

According to the incubation period, the three continuous positive wave peaks in Figures 4E,F were named P150, P200, and P250 respectively. This phenomenon in which the continuous positive wave looks like a “plateau” is different from the study of SEP in normal people and the study in this article (see Figure 4H). However, from the perspective of topographic map distribution, this research is consistent with the results in Perri et al. (2019) and the results of the contralateral healthy fingers (see Figures 4G,H), and the topographic map distribution shows a symmetrical distribution on both sides of the center.

There are not many studies on the component of P150. Zeng et al. (2006) used acupuncture to induce P150 and then traced the source to infer that the anterior cingulate cortex (ACC) was the generator of this component, while Perri et al. (2019) believed in hypnosis experiments that both the ACC and the right anterior insula are the generators of P150, and studies have shown that the anterior insula plays a key role in perceptual awareness (Craig, 2010).

Previous studies have shown that the P200 component is associated with the anterior cingulate cortex (ACC; Zeng et al., 2006), which is thought to be responsible for converting perceived stimuli into conscious perception, and the magnitude of P200 is related to the perceptual outcome of sensory processing compared to the earlier component (Lee et al., 2009).

Regarding P250, Graungaard et al. (2010) located its source in the dorsal ACC, while Perri et al. (2019) studied the medial frontal gyrus and the dorsal cingulate gyrus as generators of P250, P250 might reflect the later stage of somatosensory perception associated with affective integration of the sensory input.

In this experiment, we prefer that the positive peaks at 230 ms and 249 ms of PFM are P300 components, or that the above P250 components are integrated into P300 (Graungaard et al., 2010). The reasons are the Oddball paradigm is a classic P300 induction paradigm that could be able to induce the P300 component, the peak amplitude at 230 ms and 249 ms generated for two subjects is the largest respectively, and the peak time of 230 ms and 249 ms is the same as the latency of the P300. As for the difference in latency of different subjects, Passmore et al. (2014) showed that under some specific pathological factors, the amplitude and latency of SEP will be affected.

Different than stimuli on PFM, the stimuli on healthy finger evoke P300 that has shorter latency (204 ms for Subject 1 and 224 ms for Subject 2), and the peak amplitude declines quickly. The evoked P300 of the contralateral healthy finger has no “plateau” phenomenon (see Figures 4G,H). According to the general classification of the SEP components, the plateau can be explained that three positive peaks (P150, P200, and P300) were close and N200 disappeared. But what causes potential changes and how it affects sensory transmission in future prosthetic limbs is still a mystery. To our knowledge, P150, P200, and P300, which were endogenous components, often represent the complexity or subjects that are cognitively difficult. Based on this theory, we preliminarily infer that an amputee may have a certain degree of cognitive difficulties with ETS. However, because of the sample size and possible errors, further experimental confirmation is needed.

The main advantage of this study is to explore the components of SEP of amputees with PFM under low-frequency random stimuli. To our limited knowledge, we are the first team to study short and long latency in the ERP of ETS, which is very important for researching ETS-based sensory feedback prosthetic hands. However, the present study is not exempt from limits: for example, we were unable to perform quantitative statistical analysis because there were too few subjects; also, we did not use tools such as magnetic resonance imaging to analyze the source of the characteristics of the plateau. Individual differences between trials can also affect the results of the data. We will conduct in-depth research on this basis by recruiting more samples.

## Data Availability Statement

The original contributions presented in the study are included in the article, further inquiries can be directed to the corresponding author/s.

## Ethics Statement

The studies involving human participants were reviewed and approved by Rehabilitation Hospital Affiliated to National Research Center for Rehabilitation Technical Aids, Beijing, China. The patients/participants provided their written informed consent to participate in this study.

## Author Contributions

SB, WL, and CQ designed the work. CQ, WL, and C-HC collected and interpreted the data, wrote the initial draft and the revision of the manuscript. CQ and DX processed and analyzed the data. All authors contributed to the article and approved the submitted version.

## Conflict of Interest

The authors declare that the research was conducted in the absence of any commercial or financial relationships that could be construed as a potential conflict of interest.

## Publisher’s Note

All claims expressed in this article are solely those of the authors and do not necessarily represent those of their affiliated organizations, or those of the publisher, the editors and the reviewers. Any product that may be evaluated in this article, or claim that may be made by its manufacturer, is not guaranteed or endorsed by the publisher.
